# MAP Kinase Phosphatase-5 Deficiency Improves Endurance Exercise Capacity

**DOI:** 10.3390/cells14060410

**Published:** 2025-03-11

**Authors:** Jaime A. Perales, Ahmed Lawan, Sudip Bajpeyi, Sung Min Han, Anton M. Bennett, Kisuk Min

**Affiliations:** 1 Department of Kinesiology, University of Texas at El Paso, El Paso, TX 79968, USA; jperales7@miners.utep.edu (J.A.P.); sbajpeyi@utep.edu (S.B.); 2 Department of Biological Sciences, University of Alabama in Huntsville, Huntsville, AL 35899, USA; al0122@uah.edu; 3 Department of Physiology and Aging, University of Florida, Gainesville, FL 32610, USA; han.s@ufl.edu; 4 Department of Pharmacology, Yale University School of Medicine, New Haven, CT 06510, USA; anton.bennett@yale.edu; 5 Yale Center for Molecular and Systems Metabolism, Yale University School of Medicine, New Haven, CT 06510, USA

**Keywords:** aerobic exercise, MKP-5, mitochondrial biogenesis, cardiomyocyte proliferation

## Abstract

Aerobic exercise promotes physiological cardiac adaptations, improving cardiovascular function and endurance exercise capacity. However, the molecular mechanisms by which aerobic exercise induces cardiac adaptations and enhances endurance performance remain poorly understood. Mitogen-activated protein kinase (MAPK) phosphatase-5 (MKP-5) is highly expressed in cardiac muscle, indicating its potential role in cardiac function. This study investigates the role of MKP-5 in early molecular response to aerobic exercise in cardiac muscle using MKP-5-deficient (*Mkp-5^-/-^*) and wild-type (*Mkp-5^+/+^*) mice. Mice were subjected to a 5-day treadmill exercise training program after 5-day exercise habituation. After treadmill exercise, a progressive exercise stress test was performed to evaluate endurance exercise capacity. Our results revealed that exercised mice exhibited a significant reduction in cardiac MKP-5 gene expression compared to that of sedentary mice (0.19 ± 5.89-fold; *p* < 0.0001). *Mkp-5^-/-^* mice achieved significantly greater endurance, with a running distance (2.81 ± 169.8-fold; *p* < 0.0429) longer than *Mkp-5^+/+^* mice. Additionally, MKP-5 deficiency enhanced Akt/mTOR signaling (p-Akt/Akt: 1.29 ± 0.12-fold; *p* = 0.04; p-mTOR/mTOR: 1.59 ± 0.14-fold; *p* = 0.002) and mitochondrial biogenesis (*pgc-1α*: 1.56 ± 0.27-fold; *p* = 0.03) in cardiac muscle in response to aerobic exercise. Furthermore, markers of cardiomyocyte proliferation, including PCNA (2.24 ± 0.31-fold; *p* < 0.001), GATA4 (1.47 ± 0.10-fold; *p* < 0.001), and CITED4 (2.03 ± 0.15-fold; *p* < 0.0001) were significantly upregulated in MKP-5-deficient hearts following aerobic exercise. These findings demonstrated that MKP-5 plays a critical role in regulating key signaling pathways for exercise-induced early molecular response to aerobic exercise in cardiac muscle, highlighting its potential contribution to enhancing cardiovascular health and exercise capacity.

## 1. Introduction

The benefits of exercise or physical activity in improving health and treating disease have been well established [1,2,3]. Extensive studies have consistently shown that levels of physical activity are inversely related to all-cause mortality and cardiovascular disease mortality [4,5,6]. Specifically, aerobic exercise, also known as endurance exercise, improves cardiovascular function, reducing the risk of cardiovascular disease [7,8]. Aerobic exercise has been shown to promote physiological cardiac adaptations, including structural, functional, and molecular changes that optimize cardiac function and enhance cardiovascular health [9,10]. These adaptations facilitate more efficient transport of blood and oxygen to working muscles, thereby improving overall endurance exercise capacity. Despite the evidence supporting the positive impact of aerobic training on heart function and exercise performance, the mechanisms driving physiological cardiac adaptation to aerobic exercise have not been clearly elucidated.

The mitogen-activated protein kinase (MAPK) phosphatase-5 (MKP-5) acts as a negative regulator of MAPK signaling by dephosphorylating phosphothreonine and phosphotyrosine residues on MAPKs [11,12,13]. Recent studies have explored the involvement of MKP-5-mediated MAPK signaling in various pathological processes [12,14,15,16]. The studies have revealed that MKP-5 deficiency ameliorates the progression of muscular dystrophy and that MKP-5-deficient mice are protected from pulmonary fibrosis after lung injury [12,16]. Specifically, we have demonstrated that MKP-5 deficiency confers protection against cardiomyopathy caused by pressure overload, suggesting the importance of MKP-5 in cardiac function [15]. However, the role of MKP-5 in exercise-induced cardiac adaptation has not yet been studied. Therefore, this study aimed to test the effect of MKP-5 on early molecular response to aerobic exercise in cardiac muscle. We hypothesize that MKP-5 is a key regulator of exercise-induced early molecular change in cardiac muscle and that its deficiency enhances endurance exercise capacity.

## 2. Materials and Methods

### 2.1. Experimental Animals

8 to 10 week-old-wild type (*Mkp-5^+/+^*) and MKP-5 knockout (*Mkp-5^-/-^*) mice were used in these experiments. MKP-5 knockout mice were engineered as described previously [17]. The mice were maintained under standard conditions, including a 12 h light/dark cycle (lights on at ZT0 and off at ZT12) and ad libitum access to food and water. To minimize variability in feeding-induced signaling responses, tissues were collected at the same Zeitgeber time, and mice were not fasted before tissue collection to reflect physiological conditions. All experimental procedures involving animals were approved by the Institutional Animal Care and Use Committee of Yale University and the University of Texas at El Paso and were conducted in strict accordance with their respective guidelines.

### 2.2. Exercise Training Protocol

Prior to the initiation of exercise protocol, mice in the exercise-trained groups underwent a 5-day acclimation period on a treadmill (Columbus Instruments, Columbus, OH, USA). This acclimation involved progressively increasing both the duration and speed of the running sessions. Following a 2-day rest period, the mice ran for 60 min per day for 5 consecutive days at a speed of 15 m/min and a 0% grade. This treadmill speed has been demonstrated for young mice to maintain 70~75% of VO_2max_ [18]. A progressive exercise stress test was performed to assess endurance exercise capacity. The treadmill speed was gradually increased from 2 m/min to 6 m/min every 5 min until the point of exhaustion. Mice were considered exhausted if they failed to move forward from the back of the lane for five consecutive seconds on three separate occasions or for ten consecutive seconds once without attempting to resume running [19,20]. Endurance capacity was evaluated based on the total running distance. Mice in the sedentary group were exposed to the treadmill for the same duration and under the same conditions but without running (speed set to 0 m/min) to control for environmental and handling effects.

### 2.3. Immunoblotting

Mouse left ventricles were mechanically homogenized and lysed in lysis buffer composed of 100 mM Tris HCl (pH 7.4) and 25 mM EDTA. Protease and phosphatase inhibitors (1 mM Na_3_VO_4_, 10 mM NaF, 1 mM benzamidine, 1 mM phenylmethylsulfonyl fluoride, 1 μg/mL pepstain A, 5 μg/mL aprotinin, 5 μg/mL leupeptin) were added to the lysis buffer. The heart tissues were incubated at 4 °C for 30 min and clarified by centrifugation at 14,000 rpm at 4 °C for 10 min. The protein concentration was measured using the bicinchoninic acid (BCA) reagent according to the manufacturer’s instructions (23225; Thermo Fisher Scientific, Waltham, MA, USA). Total lysates were separated by SDS-PAGE and transferred onto nitrocellulose membranes. Membranes were blocked with 1% casein blocking reagent (1610782; Bio-Rad, Hercules, CA, USA) for 1 h at room temperature. Primary antibodies were diluted in 5% BSA in Tris-buffered saline/Tween-20 (TBST). Following overnight incubation with primary antibodies at 4 °C, membranes were washed three times for 10 min each in TBST. The membranes were then incubated with LI-COR secondary antibodies for 1 h at room temperature. The Odyssey CLx Imaging System (LI-COR Biosciences, Lincoln, NE, USA) was used to visualize and quantify antibody binding.

### 2.4. Antibodies and Reagents

All antibodies and reagents were obtained from standard chemical vendors. The antibodies used for immunoblotting are as follows. Phospho-p38 MAKP (#9215), phospho-JNK (#4668), phospho-ERK (#9101), phospho-Akt (#9271), phospho-mTOR (#2971), phospho-4E-BP1 (#9459), ERK (#9107), Akt (#2920), mTOR (#4571), p53 (#2524), and GAPDH (#2118) were purchased from Cell Signaling Technology (Danvers, MA, USA). p38 MAPK (sc-535), JNK (sc-571), 4E-BP1 (sc-9977), PCNA (cs-13110), and GATA4 (sc-25310) were sourced from Santa Cruz Biotechnology (Dallas, TX, USA). CITED4 (AV37255) was obtained from Sigma-Aldrich (Louis, MO, USA). OXPHOS antibody cocktail (MS604) was obtained from MitoSciences (Waltham, MA, USA).

### 2.5. RNA Extraction Followed by Quantitative Real-Time PCR Analysis

RNA was isolated from the mouse left ventricles using an RNeasy kit (#4104; QIAGEN, Germantown, MD, USA) following the manufacturer’s instructions. cDNA was generated from 1 μg RNA using a reverse transcriptase PCR kit (4368814; Applied Biosystems, Thermo Fisher Scientific, Waltham, MA, USA). Quantitative real-time PCR was performed using the 7500 Fast real-time PCR system (Applied Biosystems, Foster City, CA, USA). Primers for SYBR Green Master Mix (A25742; Applied Biosystems, Thermo Fisher Scientific, Waltham, MA, USA) are listed in Table 1. All relative gene expression levels were determined using the ΔΔC_T_ method, with 18S rRNA as the normalization control.

### 2.6. Statistical Analysis

All data represent the mean ± standard errors of the mean (SEM). The statistical significance between groups was determined using the two-tailed, unpaired Student’s *t*-test or two-way analysis of variance (ANOVA) followed by a Tukey post-hoc test for multiple group comparisons. Graphing and statistical analyses were performed using Prism 9 software (GraphPad, San Diego, CA, USA).

## 3. Results

### 3.1. MKP-5 Deficiency Improves Endurance Exercise Capacity in Response to Aerobic Exercise Training

**Figure 1:**
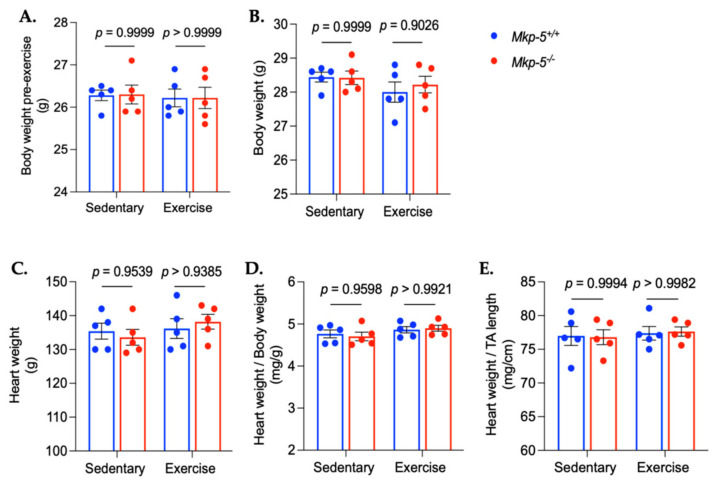
Body weight, heart weight, and cardiac morphometric ratio. Body weight pre-exercise (**A**), body weight post-exercise (**B**), heart weight (**C**), heart weight-to-body weight ratio (**D**), and heart weight-to-tibia length ratio (**E**). All data are presented as mean ± SEM. Statistical significance was assessed using 2-way ANOVA, followed by a Tukey *post-hoc* test for multiple group comparisons.

**Figure 2:**
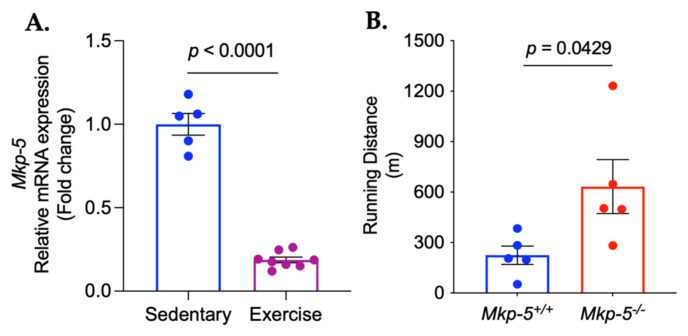
MKP-5 is downregulated in cardiac muscle in response to aerobic exercise and MKP-5 deficiency improves endurance exercise capacity. The graphs represent the relative mRNA expression of *Mkp-5* (**A**) in cardiac muscle from sedentary and exercised wild-type mice and running distance (**B**) from exercised *Mkp-5^+/+^* and *Mkp-5^-/-^* mice. All data are presented as mean ± SEM. A two-tailed, unpaired Student’s *t*-test was used for comparisons between the two groups.

Aerobic exercise improves endurance capacity by inducing various physiological and cellular adaptations in cardiac muscle [21,22]. To examine the effect of 5 consecutive days of aerobic exercise on body weight and cardiac mass, we measured body weight, heart weight, heart weight-to-body weight ratio, and heart weight-to-tibia length ratio following the exercise regimen. The results showed that five consecutive days of aerobic exercise did not lead to significant changes in either body weight or cardiac mass (Figure 1). To test whether MKP-5 gene expression is regulated in cardiac muscle in response to aerobic exercise, wild-type mice were subjected to treadmill running for five consecutive days after 5 days of exercise habituation. After aerobic exercise training, cardiac MKP-5 gene expression was reduced by 81% in the exercised mice as compared with sedentary mice (Figure 2A). This finding suggests that MKP-5 plays a role in cardiac muscle in response to aerobic exercise. Based on this result, we anticipated that MKP-5-deficient mice would have an improved ability to endure exercise. To test their exercise tolerance, the maximum exercise capacity of both *Mkp-5^+/+^* and *Mkp-5^-/-^* mice was determined by a progressive exercise stress test. The running distance of *Mkp-5^-/-^* mice was increased by 2.81-fold as compared with *Mkp-5^+/+^* mice (Figure 2B). Our findings indicate that the absence of MKP-5 enhances endurance capacity in response to aerobic exercise.

### 3.2. MKP-5 Regulates MAPKs in Cardiac Muscle in Response to Aerobic Exercise

**Figure 3:**
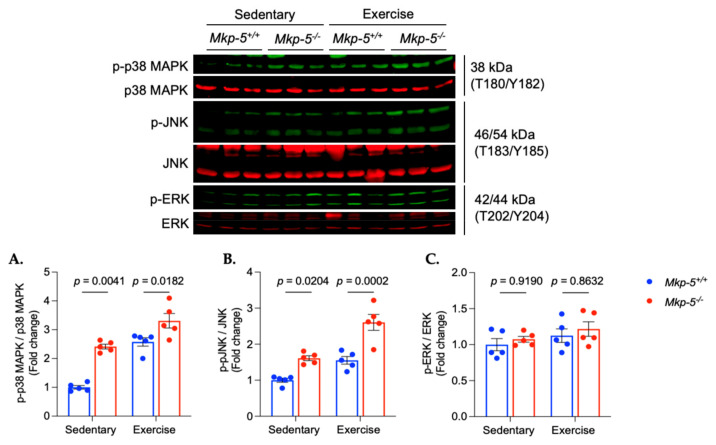
MKP-5 deficiency enhances MAPK activity in cardiac muscle in response to aerobic exercise. The graphs represent the ratio of pp38 MAPK/p38 MAPK (**A**), pJNK/JNK (**B**), and pERK/ERK (**C**) in cardiac muscle from sedentary and exercised *Mkp-5^+/+^ and Mkp-5^-/-^* mice. All data are presented as mean ± SEM. Statistical significance was assessed using 2-way ANOVA, followed by a Tukey *post-hoc* test for multiple group comparisons.

Since MKP-5 suppresses the activity of MAPKs through direct dephosphorylation, the phosphorylation of MAPKs, including p38 MAPK, JNK, and ERK, was measured in hearts isolated from sedentary and exercised *Mkp-5^+/+^* and *Mkp-5^-/-^* mice. In sedentary mice, the phosphorylation of p38 MAPK and JNK was significantly increased in the cardiac muscle of *Mkp-5^-/-^* mice as compared with *Mkp-5^+/+^* mice (Figure 3A,B). In exercised mice, the phosphorylation of p38 MAPK and JNK of *Mkp-5^+/+^* mice was increased as compared with the sedentary mice (*p* < 0.0001 and *p* < 0.0383, respectively). When compared in exercised *Mkp-5^+/+^* and *Mkp-5^-/-^* mice, the phosphorylation of p38 MAPK and JNK was significantly increased in *Mkp-5^-/-^* mice (Figure 3A,B). These results align with our previous observation that MKP-5 dephosphorylates p38 MAPK and JNK, but not ERK [12,15]. These findings indicate that MKP-5-mediated MAPK signaling is activated in cardiac muscle in response to aerobic exercise and that MKP-5 deficiency further enhances p38 MAPK and JNK in cardiac muscle from exercised mice.

### 3.3. MKP-5 Deficiency Promotes Protein Synthesis in Cardiac Muscle Following Aerobic Exercise

**Figure 4:**
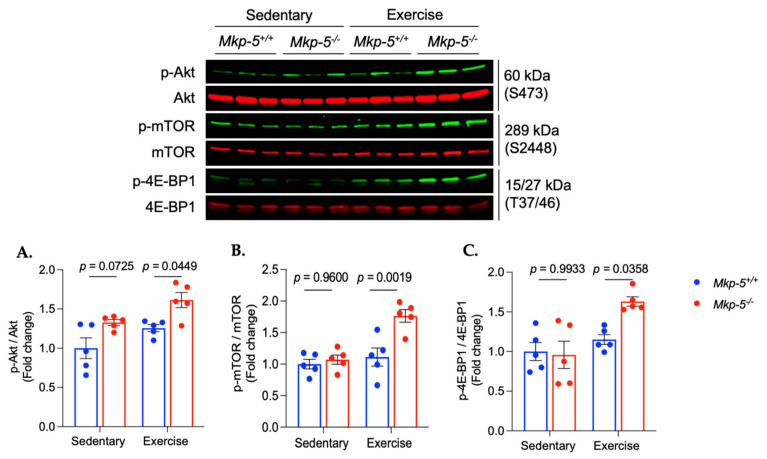
MKP-5 deficiency enhances the activation of the Akt/mTOR pathway in cardiac muscle in response to aerobic exercise. The graphs represent the ratio of pAkt/Akt (**A**), pmTOR/mTOR (**B**), and p4E-BP1/4E-BP1 (**C**) in cardiac muscle from sedentary and exercised *Mkp-5^+/+^ and Mkp-5^-/-^* mice. All data are presented as mean ± SEM. Statistical significance was assessed using 2-way ANOVA, followed by a Tukey *post-hoc* test for multiple group comparisons.

**Figure 5:**
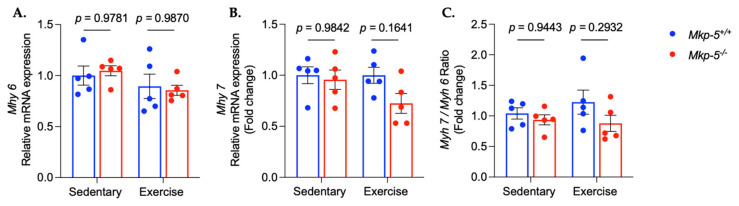
MKP-5 deficiency tends to shift myosin heavy chain isoform in cardiac muscle in response to aerobic exercise. The graphs represent the relative mRNA expression of *Myh 6* (**A**), *Myh 7* (**B**), and the ratio of *Myh 7* and *Myh 6* (**C**) in cardiac muscle from sedentary and exercised *Mkp-5^+/+^ and Mkp-5^-/-^* mice. All data are presented as mean ± SEM. Statistical significance was assessed using 2-way ANOVA, followed by a Tukey *post-hoc* test for multiple group comparisons.

Aerobic exercise has been shown to activate anabolic signaling pathways in the cardiac muscle. Specifically, growing evidence shows that the Akt/mTOR pathway contributes to cardiac physiological remodeling following aerobic exercise [23,24,25]. To test whether MKP-5 plays a role in anabolic signaling pathways in cardiac muscle in response to aerobic exercise, the activation of the Akt/mTOR pathway was measured in hearts isolated from sedentary and exercised *Mkp-5^+/+^* and *Mkp-5^-/-^* mice. MKP-5-deficient mice exhibited increased Akt phosphorylation in cardiac muscle in response to aerobic exercise as compared with *Mkp-5^+/+^* mice (Figure 4A). The phosphorylation levels of mTOR in cardiac muscle of exercised *Mkp-5^-/-^* mice were significantly increased as compared with *Mkp-5^+/+^* mice (Figure 4B). The phosphorylation of 4E-BP1, which is one of the downstream targets of mTOR, was significantly increased in cardiac muscle of mice lacking MKP-5 expression as compared with *Mkp-5^+/+^* mice (Figure 4C). The gene expression of two myosin heavy chain isoforms, *Myh 6* (myosin heavy chain α) and *Myh 7* (myosin heavy chain β), are considered molecular markers of exercise-induced cardiac adaptation (2). Pathological cardiac hypertrophy is characterized by an increase in *Myh 7* gene expression, whereas exercise-induced physiological cardiac hypertrophy is associated with a decrease in *Myh 7* gene expression [26,27,28]. To investigate the effect of MKP-5 deficiency on the alteration of *Myh 6* and *Myh 7* gene expression in the hearts of exercised mice, qRT-PCR measurement was performed in hearts isolated from sedentary and exercised *Mkp-5^+/+^* and *Mkp-5^-/-^* mice (Figure 5). In sedentary and exercised mice, relative *Myh 6* mRNA expression showed no significant differences between *Mkp-5^+/+^* and *Mkp-5^-/-^* mice. Similarly, *Myh 7* mRNA expression did not significantly differ between groups. Although there was a trend toward a reduction in *Mhy 7* mRNA expression and the *Mhy 7/Mhy 6* ratio in response to aerobic exercise in *Mkp-5^-/-^* mice, the differences did not reach statistical significance. These findings indicate that MKP-5 deficiency enhances protein synthesis in cardiac muscle following aerobic exercise, potentially contributing to improved endurance exercise capacity.

### 3.4. MKP-5 Deficiency Promotes Aerobic Exercise-Induced Mitochondrial Biogenesis

**Figure 6:**
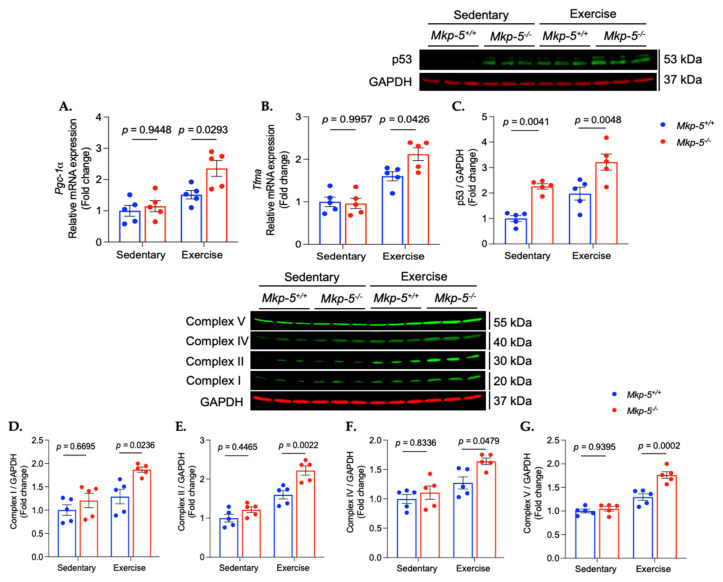
MKP-5 deficiency promotes mitochondrial biogenesis in hearts in response to aerobic exercise. The graphs represent the expression of the relative mRNA expression of *Pgc-1α* (**A**), *Tfam* (**B**), protein expression of p53 (**C**), and mitochondrial respiratory chain complexes (**D**–**G**) in cardiac muscle from sedentary and exercised *Mkp-5^+/+^ and Mkp-5^-/-^* mice. All data are presented as mean ± SEM. Statistical significance was assessed using 2-way ANOVA, followed by a Tukey *post-hoc* test for multiple group comparisons.

Mitochondrial function is necessary for the performance of endurance exercise [29,30]. It is well-established that aerobic exercise enhances mitochondrial biogenesis and respiratory function [31,32]. The process of mitochondrial biogenesis is regulated by several key factors, including the peroxisome proliferator-activated receptor gamma coactivator 1-alpha (PGC-1α), which is activated during aerobic exercise [33,34]. The activated PGC-1α promotes the expression of genes such as transcription factor A (Tfam), which is involved in mitochondrial replication and function [35,36]. In order to investigate the role of MKP-5 in cardiac mitochondrial biogenesis, the mRNA expression of *Pgc-1α* and *Tfam* was measured in cardiac muscle derived from sedentary and exercised mice (Figure 6). The cardiac muscle of *Mkp-5^-/-^* mice showed increased *Pgc-1α* and *Tfam* mRNA expression in response to aerobic exercise as compared with *Mkp-5^+/+^* mice (Figure 6A,B). Tfam is known to enhance mitochondrial biogenesis through its interaction with p53, which positively regulates mitochondrial biogenesis [37,38]. Thus, we assessed the expression of p53 in the cardiac muscle of sedentary and exercised mice. The protein expression of p53 in the cardiac muscle of *Mkp-5^-/-^* mice was significantly increased in response to aerobic exercise as compared with *Mkp-5^+/+^* mice (Figure 6C). These observations imply that MKP-5 deficiency facilitates cardiac mitochondrial biogenesis following aerobic exercise.

The increased mitochondrial biogenesis triggered by aerobic exercise activates mitochondrial oxidative phosphorylation, leading to improved ATP production, which in turn enhances endurance exercise performance [39,40]. Mitochondrial oxidative phosphorylation occurs through a series of protein complexes in the inner mitochondrial membrane [41]. To determine whether MKP-5 deficiency enhances mitochondrial protein complex expression in response to aerobic exercise, the levels of these complexes were assessed in cardiac muscle of sedentary and exercised *Mkp-5^+/+^* and *Mkp-5^-/-^* mice using immunoblotting. Aerobic exercise significantly upregulated the expression of complexes in cardiac muscles of *Mkp-5^-/-^* mice as compared with *Mkp-5^+/+^* mice (Figure 6D–G). These findings suggest that MKP-5 may play a pivotal role in optimizing endurance exercise performance through its regulation of mitochondrial function in cardiac muscle.

### 3.5. MKP-5 Deficiency Promotes Cardiomyocyte Proliferation in Response to Aerobic Exercise

**Figure 7:**
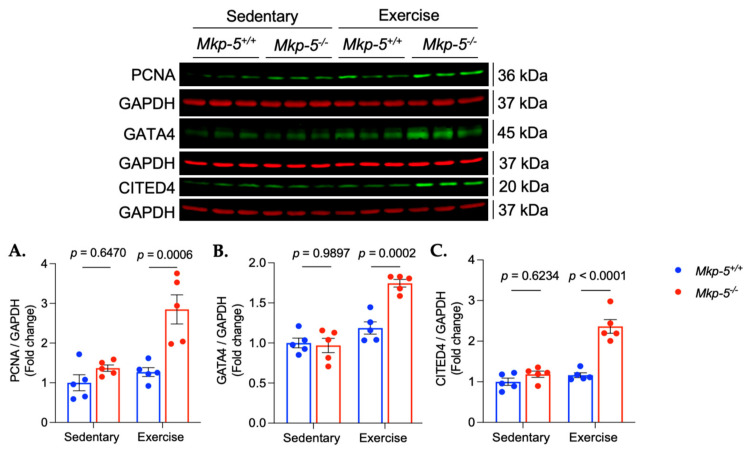
MKP-5 deficiency increases markers of cardiomyocyte proliferation in hearts in response to aerobic exercise. The graphs represent the expression of cardiomyocyte proliferation markers (**A**–**C**) in cardiac muscle from sedentary and exercised *Mkp-5^+/+^ and Mkp-5^-/-^* mice. All data are presented as mean ± SEM. Statistical significance was assessed using 2-way ANOVA, followed by a Tukey *post-hoc* test for multiple group comparisons.

One well-documented benefit of aerobic exercise is its ability to promote cardiomyocyte proliferation, which is essential for cardiac regeneration [24,42,43,44]. This process relies on anabolic pathways to support the proliferative capacity of cardiomyocytes [45,46,47]. Given that MKP-5 deficiency enhances protein synthesis pathways in the hearts of exercised mice (Figure 4), we hypothesized that MKP-5 deficiency could also facilitate cardiomyocyte proliferation in response to aerobic exercise. To test this, we first assessed cardiomyocyte proliferation using the cell proliferation marker, proliferating cell nuclear antigen (PCNA), in both sedentary and exercised *Mkp-5^+/+^ and Mkp-5^-/-^* mice. Our results demonstrated a significant increase in PCNA protein expression in hearts of exercised *Mkp-5^-/-^* mice as compared with *Mkp-5^+/+^* mice (Figure 7A). We further evaluated the expression of key transcription factors involved in cardiomyocyte proliferation, including GATA-binding protein 4 (GATA4) and Cbp/P300-interacting transactivator with Glu/Asp-rich carboxy-terminal domain 4 (CITED4) by immunoblotting. In response to aerobic exercise, *Mkp-5^-/-^* hearts exhibited increased expression of GATA4 as compared with *Mkp-5^+/+^* hearts (Figure 7B). Similarly, CITED4 expression was elevated in *Mkp-5^-/-^* hearts following exercise (Figure 7C). These findings suggest that MKP-5 deficiency may enhance exercise-induced cardiomyocyte proliferation, contributing to improved cardiac regeneration.

## 4. Discussion

In this study, we demonstrate that MKP-5 deficiency improves endurance exercise capacity. Consistent with this, MKP-5 is downregulated during exercise concomitant with the upregulation of pathways that promote protein synthesis and mitochondrial biogenesis. Further, we found that the absence of MKP-5 promotes cardiomyocyte proliferation in response to aerobic exercise. Our study suggests that MKP-5 is an important contributor to early molecular responses to aerobic exercise in cardiac muscle, thereby improving endurance exercise performance. In this study, the 5-day treadmill running protocol was chosen to specifically examine the early molecular responses to aerobic exercise in cardiac muscle. While long-term exercise is necessary for structural and functional adaptations, short-term exercise is documented to induce molecular changes that initiate the cardiac remodeling process [48,49,50]. Further, we aimed to investigate whether there was an early molecular response in cardiac muscle that could explain why MKP-5 knockout mice exhibited greater endurance capacity than wild-type mice after just 5 days of aerobic exercise. Understanding these early molecular events may provide insight into the mechanisms underlying the role of MKP-5 in exercise adaptation.

The mitogen-activated protein kinases (MAPKs) are serine/threonine protein kinases that are involved in a variety of fundamental cellular processes, including cell proliferation, differentiation, metabolic homeostasis, and cell survival [51,52]. The MAPK family includes p38 MAPK, c-Jun NH2-terminal kinases (JNKs), and extracellular signal-regulated kinases 1 and 2 (ERKs) [52]. MAPKs have been shown to be activated for cardiac adaptive responses to exercise. The level of p38 MAPK phosphorylation is increased in cardiac muscle following exercise, stabilizing the heart genome [53]. The phosphorylation of JNK is upregulated in failing hearts in response to interval treadmill exercise, improving cardiac muscle contractility [54]. Moderate-intensity aerobic exercise inhibits myocardial infarction-induced cardiomyocyte apoptosis by upregulating ERK phosphorylation in the heart muscle. This exercise-induced activation of the ERK signaling pathway ultimately improves cardiac function post-myocardial infarction [55]. These studies suggest that MAPK-mediated cellular signaling contributes to the beneficial effects of aerobic exercise on cardiac function. MAPKs are regulated through a tightly controlled multi-step process. MAPKs are activated by MAP kinase kinases (MAPKKs) via phosphorylation and deactivated by MAP kinase phosphatases (MKPs) through dephosphorylation [56]. Numerous studies have demonstrated that MAPKs are activated by MAPKKs under various cellular conditions, but the regulation of MAPKs by MKPs has often been overlooked. Given the significant reduction in MKP-5 gene expression in the hearts of exercised mice (Figure 2A), MKP-5 may be a key regulator of MAPKs in cardiac muscle in response to aerobic exercise. While this study focused on the role of MKP-5 in early molecular responses to aerobic exercise in cardiac muscle using a global knockout mouse model, it is important to acknowledge that MKP-5 in skeletal muscle may also contribute to overall exercise capacity. In a separate analysis, we found that MKP-5 was also reduced in skeletal muscle. However, the magnitude of this change was less pronounced than that observed in cardiac muscle. Given this difference, we prioritized our investigation on the more prominent cardiac muscle response.

The heart is characterized by remarkable plasticity, allowing it to adapt both structurally and functionally in response to various stimuli [57,58]. Aerobic exercise has been shown to induce physiological cardiac hypertrophy due to the increased demand for oxygen and blood flow during submaximal exercise volume [57,58]. To meet the elevated oxygen demand during aerobic exercise, the heart pumps a greater volume of blood by increasing the preload, which is the amount of blood filling the ventricles. The increased preload causes the ventricles, especially the left ventricle, to enlarge. An enlarged left ventricle increases stroke volume, which improves cardiac output, allowing more blood to be delivered to working muscles during exercise [59,60]. Aerobic exercise-induced physiological cardiac adaptations are mediated by the activation of molecular signaling pathways that promote healthy cardiac growth. One of the central pathways involved is the Akt/mTOR signaling pathway, which regulates muscle protein synthesis in response to growth factors, nutrients, and mechanical stimuli [23,25]. Akt, a serine/threonine protein kinase, phosphorylates various intracellular substrates, contributing to cell growth, metabolism, and survival [22,61]. Specifically, Akt activation leads to myocardial hypertrophy, enhancing cardiac function, while disruption of Akt signaling inhibits exercise-induced cardiac hypertrophy. DeBosch et al. demonstrated that Akt knockout mice exhibited a blunted hypertrophic response to swim training [61]. A key downstream effector of Akt is mTOR, a serine/threonine protein kinase that senses various intracellular signals, including nutrient availability and energy status [62]. In response to cellular stress, mTOR regulates critical processes such as cell growth, differentiation, autophagy, survival, and metabolism [63]. mTOR plays an essential role in exercise-induced cardiac hypertrophy, as it becomes highly phosphorylated in the myocardium following moderate endurance exercise [25,64]. A major downstream target of the mTOR pathway is 4E-BP1 which regulates mRNA translation as part of the cellular machinery responsible for protein synthesis. The activity of 4E-BP1 is inhibited by the binding of elF4E, preventing the assembly of the translation initiation complex for protein synthesis [65]. When mTOR is activated by exercise or growth factors, it phosphorylates 4E-BP1, allowing eIF4E to be released and initiate protein synthesis [66]. Although it is not well-established whether MAPKs directly phosphorylate Akt or mTOR, several studies have demonstrated that MAPKs indirectly regulate these pathways to confer cardioprotection. For instance, Hernández et al. revealed that p38 MAPK reduces the expression of the hypoxia-inducible gene REDD1 in ischemia/reperfusion (I/R) injury. This reduction leads to the dissociation of the mTOR inhibitory complex TSC1/2, thereby activating mTOR [67]. The subsequent activation of mTOR has been shown to play a cardioprotective role against I/R injury by promoting cellular survival and reducing damage [67]. Another study demonstrated that a bioactive sphingolipid suppresses apoptosis and induces anti-apoptotic autophagy in cardiomyocytes by activating the Akt/mTOR signaling pathway. However, a JNK inhibitor hampered this anti-apoptotic autophagy, suggesting that the bioactive sphingolipid could induce autophagy via phosphorylation of JNK [68]. Given our observation that MKP-5 deficiency enhances MAPK activity in cardiac muscle from exercised mice, it is plausible that MKP-5-mediated regulation of the MAPK signaling pathway contributes to the activation of the Akt/mTOR signaling pathway, thereby promoting physiological cardiac hypertrophy.

One of the most important physiological adaptations to aerobic exercise is the enhancement of mitochondrial function [31,32]. Aerobic exercise increases the demand for ATP production in working muscles, which triggers cellular signaling pathways that stimulate the creation of new mitochondria, a process known as mitochondrial biogenesis [69]. Additionally, aerobic exercise increases the activity of enzymes involved in oxidative phosphorylation, thereby making mitochondria more efficient at producing ATP to meet the energy demands of the muscles [70,71]. Mitochondrial biogenesis is induced by PGC-1α, a key transcriptional coactivator that interacts with various transcription factors, such as nuclear respiratory factors (NRFs), to promote the expression of genes involved in mitochondrial formation and function [36,72]. A wealth of evidence has demonstrated that PGC-1α can be activated by p38 MAPK in response to exercise [73]. Akimoto T. et al. showed that increased skeletal muscle activity stimulates p38 MAPK, which, in turn, enhances PGC-1α promoter activity [73]. Similarly, a study by Wright D.C. et al. revealed that swim exercise increases phosphorylation of p38 MAPK, which subsequently activates PGC-1α [74]. Once activated, PGC-1α translocates into the nucleus, where it coactivates transcription factors that regulate the expression of mitochondrial proteins [74]. One of the key targets of PGC-1α in exercise-induced mitochondrial biogenesis is Tfam, which plays an essential role in mitochondrial biogenesis by regulating mitochondrial DNA (mtDNA) replication and transcription [35,36]. Tfam binds to the promoter region of mtDNA, facilitating transcription, which is crucial for the production of the 13 proteins that form core components of mitochondrial respiratory complexes [75]. Tfam also interacts with the p53 protein to support mitochondrial biogenesis and respiratory function [37,38]. Aerobic exercise activates p53, which in turn promotes the expression of Tfam [76]. This interaction enhances mitochondrial oxidative phosphorylation, leading to improved energy production and efficiency [76]. Our data revealed that cardiac muscle from exercised mice lacking MKP-5 expression shows increased gene or protein expression of PGC-1α, Tfam, and p53, leading to enhanced expression of mitochondrial respiratory complexes (Figure 6 and Figure 7). This improvement in mitochondrial biogenesis may boost energy production capacity, thereby enhancing the endurance exercise capacity of MKP-5 deficient mice.

The relationship between aerobic exercise and cardiomyocyte proliferation has become a topic of increasing interest in cardiovascular research. Cardiomyocytes play a critical role in heart regeneration and repair [77]. It was believed that adult cardiomyocytes had a very limited capacity for regeneration and proliferation. However, recent studies suggest that certain conditions, including aerobic exercise, may promote this process. One of the key regulators of cardiomyocyte proliferation is GATA4, a transcription factor that has been reported to mark proliferative cells during cardiac regeneration [78]. Evidence indicates that GATA4 not only regulates cardiomyocyte proliferation but is also involved in the cardiac adaptive response to exercise. Broderick et al. demonstrated that treadmill exercise significantly increases both mRNA and protein expression of GATA4 in cardiac muscle [79,80]. This finding highlights the role of GATA4 in exercise-induced cardiac remodeling. Another important transcription factor involved in cardiomyocyte proliferation is CITED4, which plays a critical role in exercise-induced cardiac hypertrophy [43,44,80]. Boström et al. found that 14 days of aerobic exercise robustly increases CITED4 expression in cardiac muscle, further promoting cardiomyocyte proliferation [43]. The activation of both GATA4 and CITED4 is negatively regulated by CCAAT/enhancer-binding protein beta (C/EBPβ), which acts as a suppressor of cardiomyocyte proliferation [43]. Emerging evidence suggests that exercise activates Akt, which inhibits C/EBPβ. This inhibition of C/EBPβ removes its repressive effects, allowing for increased transcription of GATA4 and CITED4. The subsequent activation of CITED4 leads to the phosphorylation of mTOR, a key regulator of cell growth, thereby promoting exercise-induced cardiomyocyte proliferation and cardiac hypertrophy [81]. Consistent with this pathway, our data demonstrate that, in response to aerobic exercise, MKP-5 deficiency activated the Akt/mTOR axis, which may subsequently upregulate GATA4 and CITED4, contributing to cardiomyocyte proliferation.

## 5. Limitations and Future Directions of Experimental Approach

While our study provides valuable insights into the molecular mechanisms underlying exercise-induced early molecular responses to aerobic exercise, we acknowledge several limitations in our experimental approach that warrant consideration.

The exercise protocol utilized in this study consisted of a 5-day treadmill running regimen at moderate intensity following a 5-day treadmill running habituation period. This short-term duration was selected to examine the early molecular responses to aerobic exercise, particularly the activation of signaling pathways involved in cardiac adaptation. However, it may not fully reflect long-term structural and functional cardiac adaptations. Additionally, since this study focused on the effects of short-term aerobic exercise on the early molecular responses in cardiac muscles, functional assessments, such as VO_2_max and echocardiogram, were not included in this study. To expand upon our findings, future studies will examine the role of MKP-5 in long-term aerobic exercise-induced chronic cardiac adaptations, allowing us to determine whether its deficiency contributes to sustained structural and functional remodeling. Further, future studies will use an MKP-5 cardiac-specific knockout model to isolate the direct role of MKP-5 in cardiac muscle. We will also determine whether exercise-induced MKP-5 modulation provides cardioprotection against various cardiomyopathy models.

## 6. Conclusions

Our findings demonstrated that MKP-5 is involved in mediating the effects of aerobic exercise on improving endurance performance. Specifically, MKP-5 deficiency promotes protein synthesis, mitochondrial biogenesis, and cardiomyocyte proliferation, contributing to improved exercise-induced early molecular cardiac adaptations and endurance exercise capacity. These results highlight the critical role of MKP-5 in mediating physiological cardiac remodeling and suggest its potential as a therapeutic target for improving cardiac performance in response to exercise.

## Figures and Tables

**Table 1:** The list of primer sequences used for quantitative real-time PCR analysis.

Primer Name	Sequences
*Mkp-5*	5′-ACCGCAGCTAGGAATAATGGA-3′ 5′-ACCAAAAGCCTTGACTCCG-3′
*Pgc-1* *α*	5′-CCCTGCCATTGTTAAGACC-3′
	5′-CTTTTGTCCTTGTCGTCGTC-3′
*Tfam*	5′-ATTCCGAAGTGTTTTTCCAGCA-3′ 5′-TCTGAAAGTTTTGCATCTGGGT-3′
*Myh6*	5′-GTCCCGGACACTGGACCAGGCC-3′ 5′-CTCCTTTTCTTCCAGTTGCCTAGCCAA-3′
*Myh7*	5′-GAGCAAGGCCGAGGAGACGCAGCGT-3′ 5′-GAGCCTCCTTCTCGTCCAGCTGCCGG-3′
*18S*	5′-ACCGCAGCTAGGAATAATGGA-3′ 5′-ACCAAAAGCCTTGACTCCG-3′

## Data Availability

The data presented in this study are available on request from the corresponding author.
